# Hereditary Nephrogenic Diabetes Insipidus: Pathophysiology and Possible Treatment. An Update

**DOI:** 10.3390/ijms18112385

**Published:** 2017-11-10

**Authors:** Serena Milano, Monica Carmosino, Andrea Gerbino, Maria Svelto, Giuseppe Procino

**Affiliations:** 1 Department of Biosciences, Biotechnologies and Biopharmaceutics, University of Bari, 70126 Bari, Italy; serena.milano@uniba.it (S.M.); andrea.gerbino@uniba.it (A.G.); maria.svelto@uniba.it (M.S.); 2 Department of Sciences, University of Basilicata, 85100 Potenza, Italy; monica.carmosino@unibas.it

**Keywords:** nephrogenic diabetes insipidus (NDI), aquaporin-2 (AQP2), arginine-vasopressin (AVP), arginine-vasopressin receptor AVPR2, antidiuresis, polyuria

## Abstract

Under physiological conditions, excessive loss of water through the urine is prevented by the release of the antidiuretic hormone arginine-vasopressin (AVP) from the posterior pituitary. In the kidney, AVP elicits a number of cellular responses, which converge on increasing the osmotic reabsorption of water in the collecting duct. One of the key events triggered by the binding of AVP to its type-2 receptor (AVPR2) is the exocytosis of the water channel aquaporin 2 (AQP2) at the apical membrane the principal cells of the collecting duct. Mutations of either AVPR2 or AQP2 result in a genetic disease known as nephrogenic diabetes insipidus, which is characterized by the lack of responsiveness of the collecting duct to the antidiuretic action of AVP. The affected subject, being incapable of concentrating the urine, presents marked polyuria and compensatory polydipsia and is constantly at risk of severe dehydration. The molecular bases of the disease are fully uncovered, as well as the genetic or clinical tests for a prompt diagnosis of the disease in newborns. A real cure for nephrogenic diabetes insipidus (NDI) is still missing, and the main symptoms of the disease are handled with s continuous supply of water, a restrictive diet, and nonspecific drugs. Unfortunately, the current therapeutic options are limited and only partially beneficial. Further investigation in vitro or using the available animal models of the disease, combined with clinical trials, will eventually lead to the identification of one or more targeted strategies that will improve or replace the current conventional therapy and grant NDI patients a better quality of life. Here we provide an updated overview of the genetic defects causing NDI, the most recent strategies under investigation for rescuing the activity of mutated AVPR2 or AQP2, or for bypassing defective AVPR2 signaling and restoring AQP2 plasma membrane expression.

## 1. Introduction

Tight regulation of water homeostasis is essential for most physiological processes in all living organisms. The evolution of mammals has selected different homeostatic mechanisms that work together to maintain body-fluid osmolality at approximately 300 mosm/kg by regulating the intake or excretion of water and salt. Among these mechanisms, urinary excretion of water is finely regulated to allow a rapid adaptation to water uptakes and losses and maintain constant salt concentration in intra and extracellular fluids.

Under physiological conditions, the production of urine is obligatory. Blood is constantly filtered through the glomerular barrier, which behaves like an anatomically precise ‘bandpass filter’ [1] and produces up to 180 L/day of pro-urine within the Bowman’s capsule. However, only less than 1% of this large initial volume will be excreted, by virtue of the massive water reabsorption taking place in the renal tubule. Proximal tubules and the Henle’s loops are responsible for obligatorily reabsorbing approximately 90% of the water composing the filtered load through the water channel aquaporin 1 (AQP1) [2]. The remaining 18 to 20 L of the forming urine reaching the distal tubules and the collecting ducts are subjected to regulated water reabsorption depending upon plasma osmolality and blood volume, thus defining the final urine volume to be excreted (1.5 to 2 L/24 h).

**Figure 1:**
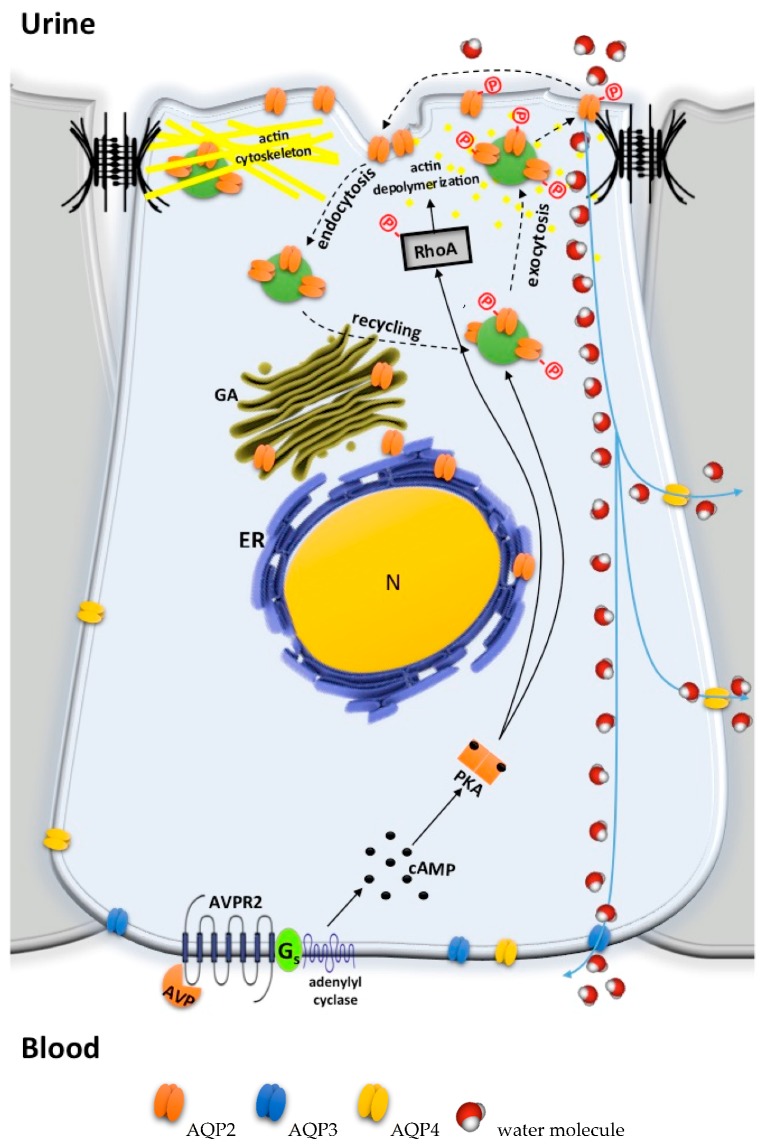
Action of arginine vasopressin (AVP) in the collecting duct principal cells. Upon the binding of AVP to its cognate receptor AVPR2 at the basolateral membrane, a stimulatory G protein α subunit (G_s_) activates adenylyl cyclase and increases cyclic adenosine monophosphate (cAMP) intracellular concentrations. This, in turn, activates protein kinase A (PKA), which phosphorylates many substrates, including AQP2 and RhoA (full lines). The partial depolymerization of the sub-apical actin cytoskeleton facilitates the apical exocytosis of AQP2-storage vesicles (dotted lines). Water enters the cells via de novo inserted AQP2 tetramers at the apical membrane and leaves the epithelial cells through AQP3 and AQP4 constitutively expressed at the basolateral membrane. AVP removal from the bloodstream allows AQP2 endocytosis and recycling through early endosomes (dotted lines).

Increases in plasma osmolality (hypernatremia) or decreases of the blood volume (hypovolemia), occurring during water deprivation, signal to the kidneys to conserve water, a physiological condition known as antidiuresis. In particular, shrinkage of hypothalamic osmoreceptors or decreased activity of aortic and carotid baroreceptors [3] triggers the release of the antidiuretic hormone arginine vasopressin (AVP) from the posterior pituitary into the bloodstream [4,5]. The antidiuretic action of AVP (Figure 1) begins upon binding to type-2 vasopressin receptor (AVPR2) [6], a G protein-coupled receptor localized at the basolateral plasma membrane of the principal cells of the kidney collecting duct (Figure 1). Once activated by AVP, AVPR2 initiates a signal transduction cascade that consists of the activation of adenylate cyclase (AC) via the stimulatory G (Gs) protein, an increase in intracellular cyclic adenosine monophosphate (cAMP) concentration, and the activation of protein kinase A (PKA). A crucial step in this process is RhoA inhibition and partial depolymerization of subapical actin cytoskeleton [7]. As a final result, the water channel aquaporin 2 (AQP2) is phosphorylated and translocated from a pool of intracellular storage vesicles to the apical plasma membrane of the principal cells [8], highly increasing water permeability at this site [9]. The osmotic gradient generated by reabsorption of NaCl in the medullary thick ascending limb (TAL) and urea in the medullary collecting duct, both also regulated by AVP [10], provides the driving force for AQP2-mediated water reabsorption within the principal cells. The exit pathway for water entering the cells is represented by aquaporin 3 and 4 (AQP3/4), expressed at the basolateral membrane of the same cells mediating water flux to the extracellular fluid and ultimately to the blood. This process restores plasma osmolality and volume and is regulated by a negative feedback. In fact, upon the restoration of water balance, AVP release from the pituitary ceases and AQP2 is sequestered into recycling endosomes within the cells [11], thus reducing the water permeability of the collecting duct. In humans, during antidiuresis, AVP increases urine osmolality up to 1200 mosm/kg and decreases urine flow. On the other hand, AVP removal from the bloodstream or AVPR2 desensitization/internalization [12] restores diuresis, characterized by low urine osmolality (below 50 mosm/kg) and higher urine output [10].

Previous studies have demonstrated that plasma AVP levels begin to fall within minutes of water consumption, even prior to changes in blood osmolality, underlying the anticipatory regulation of AVP neurons [13,14,15]. The recent development of methods for optically recording deep brain calcium dynamics has made it possible to measure for the first time how these neurons are regulated during behaviour [16,17,18].

Recently, Mandelblat-Cerf and colleagues, using electrophysiological and optical methods, demonstrated that the ingestion of water leads to a rapid presystemic decrease in the activity of AVP neurons in the supraoptic nucleus [16]. In addition, fibre photometry recordings revealed that glutamatergic neurons in the subfornical organ are rapidly inhibited during drinking to coordinate the anticipatory control of thirst and AVP secretion [17]. Furthermore, Zocchi et al. demonstrated that acid-sensing taste receptor cells mediate the taste response to water [19].

The rapid inhibition of AVP neurons explains why circulating levels of AVP begin to decline at the outset of drinking, in anticipation of the restoration of homeostasis [18].

It is intuitive to understand how defects in the production/release of AVP, the reduced affinity of AVPR2 to AVP, and the altered trafficking of AQP2 dramatically impair water reabsorption in the kidney. The congenital form of nephrogenic diabetes insipidus (NDI) is a rare inherited disorder, characterized by insensitivity of the distal nephron to the antidiuretic action of AVP and the reduced ability of the kidney to concentrate the urine, leading to severe dehydration and electrolyte imbalance (hypernatremia and hyperchloremia). In most cases, NDI is caused by a non-functional AVPR2 receptor (X-linked NDI). Mutations of the AQP2 gene also lead to congenital NDI. 

Frequently, NDI can be acquired in adulthood as a side effect of pharmacological treatments with lithium [20], drugs [21], and antibiotics/antifungal/antiviral [22,23,24,25,26,27]. Hypokalemia or hypercalcemia/hypercalciuria also cause acquired NDI [28,29,30,31,32], as well as acute and chronic renal failure (ARF, CRF) [33,34,35,36,37,38]. In some cases, the downregulation of AQP2 expression or altered trafficking are responsible for acquired NDI. In this review, we will focus our attention on the genetic defects leading to congenital NDI.

## 2. Pathophysiology of Congenital Nephrogenic Diabetes Insipidus

The main clinical hallmarks of NDI are polyuria and compensatory polydipsia. Upon inadequate water supply, a hot environment, or episodic losses of free water, patients suffering from NDI do not properly compensate water loss and are at risk of severe dehydration.

The urine concentrating defect is present at birth, and symptoms arise during the first week of life as irritability, poor feeding, and failure to thrive [39]. Some signs of dehydration are dryness of the skin, loss of normal skin turgor, recessed eyeballs, increased periorbital folding, and depressed anterior fontanel. Upon initiation of pharmacological treatment (see below), most recover their weight loss [40]. Persistent polyuria can lead to the development of kidney megacystis, trabeculated bladder, hydroureter, and hydronephrosis [39].

Repeated episodes of dehydration can cause mental retardation [41,42], which is a serious complication of NDI [43], probably secondary to hypoxic episodes. However, if diagnosed early and treated, mental retardation is rare in NDI patients. Nevertheless, the psychological development of these patients is adversely affected by the persistent need to drink and frequent voiding.

The clinical diagnosis of NDI relies on the demonstration of a reduced ability to concentrate the urine, despite the presence of high plasma AVP or the parenteral administration of AVP or desmopressin (DDAVP^®^) [40]. To confirm/establish the diagnosis in a proband, male or female, genetic testing is performed on the AVPR2 gene by sequencing and deletion/duplication analysis. AQP2 gene sequencing is performed first in cases of affected children from consanguineous parents. Only if pathogenic variants of AQP2 are not identified, AVPR2 sequencing is performed. Congenital NDI is caused by mutations in the AVPR2 or the AQP2 genes. Three different inheritance patterns of NDI have been recognized. In most cases (about 90%), NDI is transmitted as an X-linked recessive trait (MIM #304800) [44], caused by mutations in the AVPR2 receptor gene located on the X chromosome at locus Xq28 [45]. A minority of patients (about 10%) show an autosomal recessive (MIM #222000) or dominant trait (MIM #125800) as a result of mutations in the AQP2 gene, located on chromosome 12q13 [46].

### 2.1. AVPR2 Mutations Leading to X-Linked NDI

The AVPR2 [47] is a typical seven membrane-spanning helices G protein-coupled receptor (GPCR). Mutations in the AVPR2 gene lead to X-linked NDI (X-NDI) [44].

X-NDI male patients do not concentrate urine, even after the administration of exogenous AVP [48]. In contrast, heterozygous females, due to skewed X-chromosome inactivation, show variable degrees of polyuria and polydipsia [49,50,51]. Depending on the position of the mutation, a partial phenotype can be seen in some patients [52]. The number of identified AVPR2 mutations leading to X-NDI is constantly increasing. As of September 2017, according to the Human Gene Mutation Database, 274 identified mutations of AVPR2 gene are classified as ‘loss of function’ (see Table 1). AVPR2 mutations have been classified into five distinct groups according to sequence analysis and subcellular localization [53].

Class I mutations interfere with proper transcription, mRNA processing, and translation, leading to truncated proteins [54], which are often rapidly degraded.

**Figure 2:**
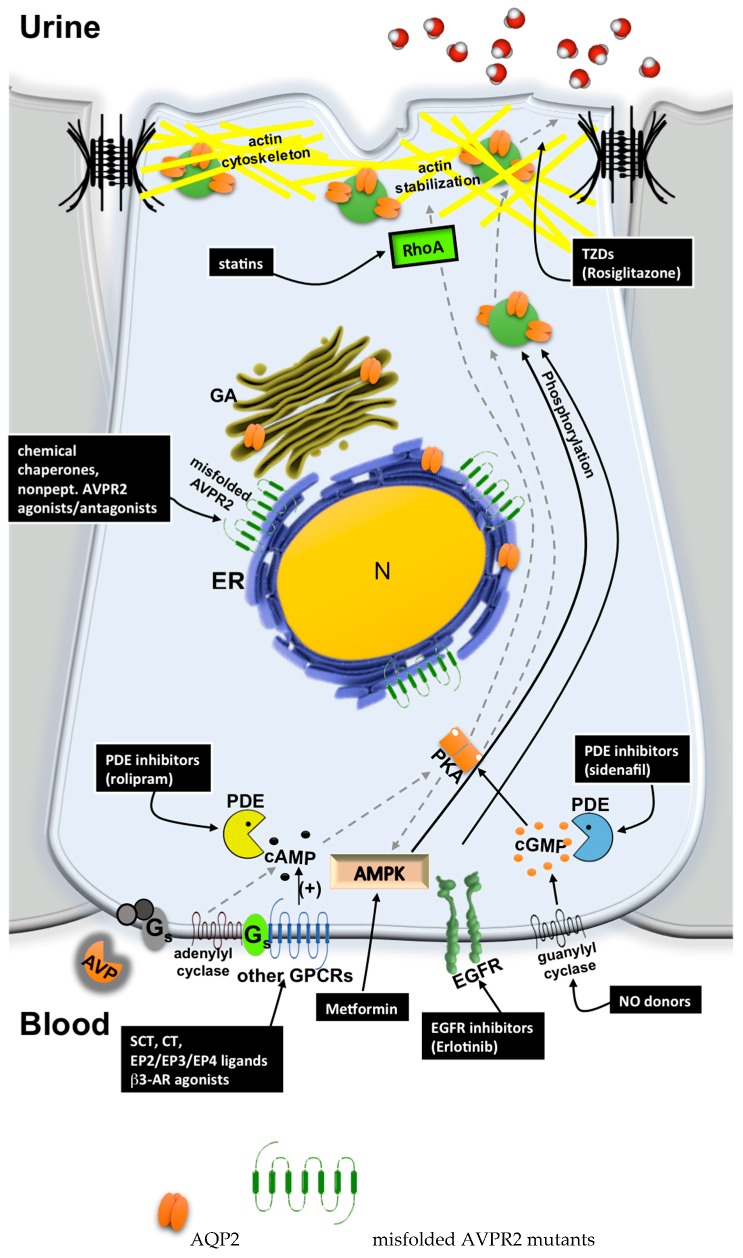
The current possible strategies to bypass AVPR2 mutations responsible for X-linked NDI. The most recurrent mutations of AVPR2 responsible for X-linked NDI are class II mutations producing full-length misfolded proteins trapped in the endoplasmic reticulum (ER), although retaining intrinsic functionality. A lack of AVPR2 basolateral expression prevents AVP signaling and AQP2 exocytosis (grey dotted lines). Possible therapeutic strategies (full lines) for treating X-linked NDI are focused on: (1) the use of chemical chaperones aiding protein folding and inducing export from the ER; nonpeptide AVPR2 antagonists, like vaptans, that stabilize receptor conformation in the ER, thus allowing it to bypass the ER quality control mechanism; and nonpeptide AVPR2 agonists that promote interaction with adenylyl cyclase, thus increasing cAMP concentration (+). (2) Activation of the cAMP pathway by stimulating other G proteins-coupled receptors (GPCRs) coupled to Gs/adenylyl cyclase expressed in collecting duct principal cells such as secretin (SCT), calcitonin (CT), E-prostanoid receptors (EP2/EP3/EP4) and β3-adrenoreceptor; the inhibition of phosphodiestherases (PDE) to increase basal cAMP levels (+). (3) The activation of the cyclic guanosine monophosphate (cGMP) pathway, promoting AQP2 exocytosis either by stimulating guanylyl cyclase or by inhibiting PDE. (4) The inhibition of epidermal growth factor receptor (EGFR), which counteracts AVP-mediated AQP2 exocytosis by a not fully elucidated mechanism. (5) The activation of AMP-activated protein kinase (AMPK) promoting AVP-independent AQP2 phosphorylation. (6) Thiazolidinediones (Rosiglitazone) likely promotes Ca^2+^ influx that triggers AQP2 exocytosis at the plasma membrane in the absence of AQP2 phosphorylation. (7) Statins treatment that inhibits RhoA, promotes cortical actin depolymerization, and facilitates the constitutive exocytosis of AQP2 at the apical membrane.

Class II mutations, the most common, are missense or insertion/deletion producing full-length misfolded proteins, mostly retained in the endoplasmic reticulum (ER) by the ER quality-control machinery and targeted for proteasome degradation [55] (Figure 2).

Class III mutants result in plasma membrane-expressed receptors with reduced affinity for the stimulatory Gs protein, leading to blunted activation of the phosphorylation pathway, promoting AQP2 exocytosis [54]. Class IV mutants have low affinity for vasopressin [54]. Class V mutants are misrouted to different subcellular organelles like arrestins-positive endocytic vesicles [56,57].

Conversely, AVPR2 can also be affected by ‘gain of function’ mutations. These mutant receptors have increased binding affinity to AVP [58] or are constitutively activated, causing the nephrogenic syndrome of inappropriate antidiuresis (NSIAD) [59,60,61].

In addition, large deletions of the AVPR2 gene may also encompass the L1CAM gene, mapping adjacent to the AVPR2 [62], and be responsible for both X-NDI and hydrocephalus [63,64,65]. However, isolated point mutations in the L1CAM gene are not associated with X-NDI [66]. Partial NDI, probably secondary to cystic dysplastic kidneys, was also reported in a 17-year-old boy carrying a mutation of the HNF1B gene [67].

### 2.2. AQP2 Mutations Leading to Autosomal Recessive/Dominant NDI

The AQP2 gene is located on chromosome 12q13 and codes for the 271 amino acid AQP2 protein, a type IV-A transmembrane protein characterized by six transmembrane domains connected by five loops and intracellular N- and C-termini [68].

Phosphorylation at serine 256 is required for cAMP-dependent regulatory exocytosis of the AQP2 [8]. Studies in vitro and in vivo strongly suggest a role for both S256 and S269 in the membrane accumulation of AQP2 [69,70,71]. With respect to other phosphorylation sites (S261 and S264), recent studies suggest that these sites play minimal roles in AQP2 plasma membrane targeting [72,73].

About 10% of NDI patients are affected by an autosomal form of NDI. Similar to the AVPR2 inactivating mutations, those on AQP2 can affect the proper synthesis, processing, or plasma membrane localization of the gene product, thus preventing the antidiuretic action of AVP in the collecting duct principal cells [48].

**Figure 3:**
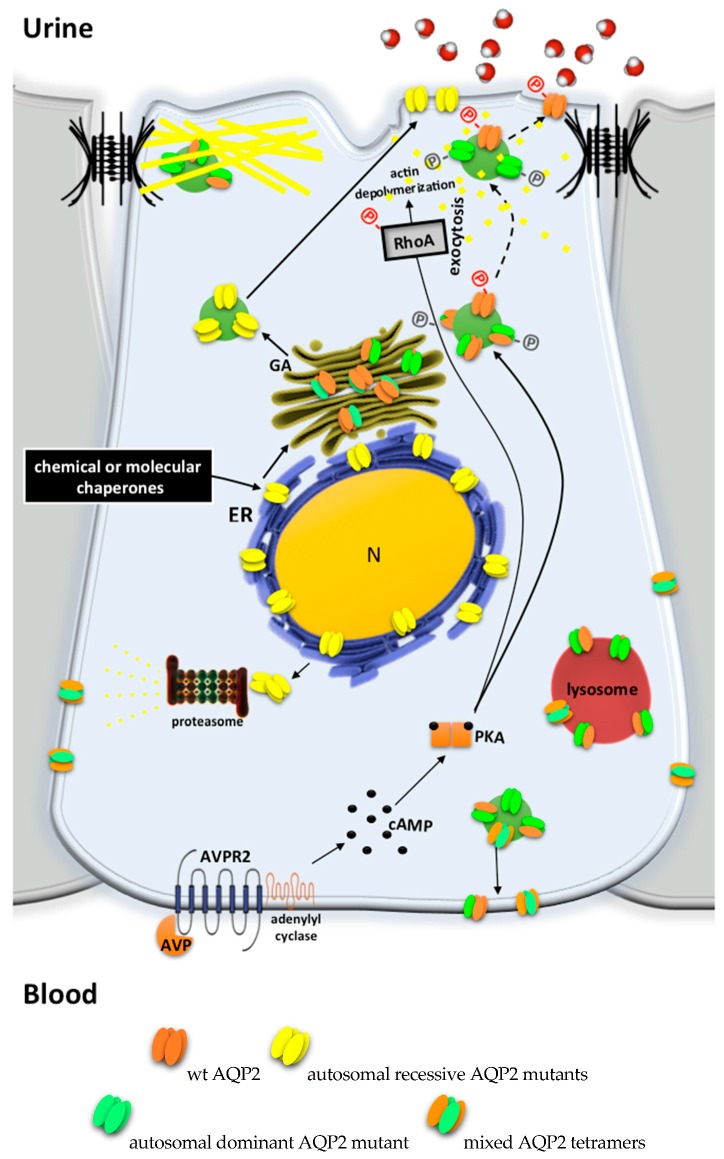
AQP2 mutations explain autosomal recessive and dominant NDI. AQP2 mutations can affect the proper synthesis, processing or plasma membrane localization of the gene product. Most of the AQP2 mutations falling in the protein transmembrane domains are misfolded (yellow tetramers) and retained in the ER until degraded by the proteasome. Affected patients are homozygous or compound heterozygous for these AQP2 mutations. Since most of these mutants still maintain water channel functionality, the therapeutic approach under investigation is based on the use of chemical chaperones aiding release from the ER (full lines). Autosomal dominant NDI is caused by AQP2 mutations affecting the carboxyl terminus (COOH-terminus) of the protein, which is a crucial domain for phosphorylation or apical sorting. A lack of AQP2 exocytosis (dotted lines) prevent the AVP-mediated water reabsorption in the collecting duct principal cells.These AQP2 mutants have a dominant effect over the wtAQP2 subunit and are responsible for AQP2 missorting.

Currently, 65 mutations of the AQP2 gene have been described as causative of autosomal NDI, most of which show a recessive inheritance (see Table 1). Patients are homozygous or compound heterozygous for these AQP2 mutations. Mostly missense, these mutations affect aminoacids in the AQP2 transmembrane domains, resulting in protein misfolding. ER accumulation of these AQP2 mutants has been shown in several studies [74,75,76,77,78] (Figure 3). As for AVPR2, ER retention due to extended chaperone interaction eventually leads to proteasome degradation. However, some mutants retain intrinsic functionality as water channels and show partial activity when expressed in the apical membrane by means of forced transport or overexpression [77] or by ectopic expression in *Xenopus* oocytes [79], indicating that the native conformation is only slightly disturbed. This evidence suggests that the disease phenotype is due to aberrant subcellular localization of AQP2 rather than a loss of function. This is of great therapeutic significance for restoring the trafficking of these mutants.

A small number of AQP2 mutations (11 out of 65) are inherited in a dominant trait and are causative of autosomal dominant NDI [80,81,82,83]. These mutations affect aminoacids at the carboxyl-terminal of AQP2 containing regulatory sequences for trafficking and sorting. The heterotetramers formed by WT and mutated AQP2 monomers are either retained in the Golgi apparatus [83,84] or are misrouted to late endosomes, lysosomes [77], or basolateral membrane [85] (Figure 3). 

### 2.3. Partial NDI

The majority of patients with X-NDI display little or no rise in urine osmolality in response to fluid deprivation tests or large doses of AVP or desmopressin (DDAVP^®^). Nevertheless, a few patients have been reported to concentrate their urine quite efficiently during fluid deprivation tests or infusion with AVP or DDAVP [86,87]. This residual urine concentrating ability may protect against episodes of severe hypertonic dehydration, to which patients with severe defects are susceptible. The age of onset of the disease in individuals with partial X-linked NDI usually appears later in life. Up to the point of our Medline search, only 17 of all known missense mutations identified in the AVPR2 gene have been associated with the partial X-NDI phenotype [87,88].

Interestingly, in the majority of AQP2 mutations causing autosomal dominant NDI, AQP2 mutants retain residual trafficking to the apical membrane in response to AVP, thus resulting in a less severe concentrating defect (partial NDI). This is supposedly due to the fact that one-sixteenth of all tetramers formed in dominant NDI are wt-AQP2-only tetramers [89,90] (Figure 3).

In addition to genetic defects, partial NDI may be also attributable to aging. It has been reported that, in both humans and rats, the aging results in a reduced maximal urine concentrating ability because of the downregulation of AQP2 and urea transporters [91,92].

## 3. Animal Models to Study NDI

A variety of mouse models of NDI have been developed over the years. The generation of NDI transgenic mice indisputably improved the understanding of AQP2 and AVPR2 roles in water and salts homeostasis in health and disease.

Transgenic mouse models for NDI are useful to elucidate potential compensatory or adaptive changes in the kidney and to examine novel potential therapeutic strategies targeting specific AQP2 and AVPR2 mutations in order to correct/minimize the urine-concentrating defect. It must also be mentioned that the deletion or mutation of several other genes can result in severe defects in the ability to concentrate urine and in the resistance of the kidney to AVP, suggesting an ‘NDI-like phenotype’ [93,94,95].

### 3.1. Models of Autosomal Recessive NDI

Several models for autosomal recessive NDI have been generated. The first animal model of renal AQP2 deficiency/mutation was proposed by Yang et al., which created knock-in mice carrying the recessive AQP2-T126M mutation [96]. The mutant mice appeared normal at two to three days after birth but died within day 6 after birth, indicting a critical role of AQP2 in neonatal renal function [96]. 

Rojek and colleagues, generated AQP2-total-KO mice, as well as mice expressing AQP2 exclusively in the connecting tubule (CNT) and not in the collecting duct (AQP2-CD-KO) [97]. AQP2-total-KO mice died postnatally (days 5 to 12) because of an excessive loss of extracellular fluid volume. In contrast, the AQP2-CD-KO mice survived to adulthood, despite a severe urinary concentration defect [97]. These findings suggest that AQP2 expressed in the CNT is fundamental to the rescue of the lethal phenotype observed in total AQP2 knockout mice and that it cannot be compensated for by other mechanisms. In AQP2-CD-KO mice, water restriction caused only a slight decrease in urine output with no change in urine osmolality, revealing the absence of compensatory mechanisms and the importance of AQP2 function in water balance regulation in the CD [97].

A spontaneous mutation responsible for autosomal recessive traits has also been identified by McDill et al., which described a single base change in codon 256 of AQP2 [98]. This mutation causes the substitution of serine 256 (S256) by a leucine, thus preventing AQP2 phosphorylation at S256 and resulting in basolateral distribution. Mutant S256L had no response to DDAVP, produced large volumes of hypotonic urine, and showed severe symptoms of both hydronephrosis and obstructive nephropathy. About 90% of mutant mice died between two and four weeks of age [98].

Lloyd reported the characterization of a mouse model with a homozygous F204V mutation in the AQP2 gene [75]. F204V mice survived to adulthood, grew, and reproduced normally. It is the first mouse model of NDI to survive to maturity. Similar to other recessive mutations of AQP2, F204V mutant protein was mainly retained in the ER. The smaller response to DDAVP indicated that AQP2 F204V had only a limited misfolding and may retain some residual trafficking activity, which must be sufficient to allow the survival of the individual. In heterozygous mice, Lloyd et al. additionally showed that AQP2-F204V monomers can homotetramerize as well as form heterotetramers with wt-AQP2.

Heterozygous mice showed no NDI symptoms, and AQP2 localized at luminal membrane after DDAVP infusion as in wild type [75]. Therefore, the degree of AQP2 misfolding and the possibility of this mutant overcoming ER quality control define the severity of the recessive NDI phenotype.

Furthermore, mice with deleted distal C-terminal tails of the AQP2, including S256, but still retaining the putative apical localization signal (AA 221–229), were generated [99]. Homozygous mice survived to adulthood if water was administrated at the time of weaning; they showed increased urine output and decreased urine osmolality and were resistant to DDAVP injection [99].

Next, inducible knock-in AQP2-T126M mice were generated [100]. Mutant mice developed polyuria and urine concentration defects and were used to test a putative small molecule corrector in vivo [100].

### 3.2. Models of Autosomal Dominant NDI 

To investigate the pathogenesis of dominant-type NDI and to test the potential efficacy of the treatment with Rolipram, Sohara et al. generated a mutant AQP2 (763–772 del) knock-in mouse [81]. The heterozygous mutant AQP2 (763–772 del) knock-in mice manifested a severe urine-concentrating defect, although it was milder than that in autosomal-recessive NDI T126M AQP2 knock-in mice [96]. They survived without supplemental fluid and, after water deprivation or DDAVP injection, showed increased urine osmolality. 

Immunofluorescence analysis of dehydrated animals revealed that AQP2 was mainly missorted to the basolateral membrane, although a weak staining of wild-type AQP2 was also detected at the apical plasma membrane, thus explaining the renal phenotype of these mice [81]. 

### 3.3. Models of X-Linked NDI

Yun et al. created a mouse model lacking functional expression of AVPR2 by introducing into the mouse genome a nonsense mutation known to cause X-NDI in humans (E242X) [101].

AVPR2-deficient male mice were polyuric at birth, failed to thrive, and died within the first week after birth due to hypernatremia and dehydration caused by the inability of these animals to concentrate their urine [101]. Transcriptome-wide screening of renal and hypothalamic gene expression in AVPR2-deficient mice [101] was performed by Schliebe et al. [102].

The expression of several genes involved in sodium and water reabsorption was upregulated in the kidneys of three-day-old male AVPR2-deficient mice, including AQP1, carbonic anhydrases, Na^+^-K^+^-ATPase, NaCl and HCO3^−^ transporters, and cyclooxygenase 2, suggesting compensatory changes [102]. Despite these compensatory changes, E242X mice died within one week, making them an unsuitable model for studying X-NDI in adult mice.

Recently, the first viable mouse model of X-NDI in which the AVPR2 gene can be conditionally deleted during adulthood by tamoxifen administration has been generated [103].

After AVPR2 deletion, adult mice displayed all key symptoms of X-NDI, including polyuria, polydipsia, urine hypo-osmolality, and resistance to AVP [103]. 

Another tool to study AVPR2 functions, compensatory mechanisms due to AVPR2 inactivation, and potential pharmacological therapies for the treatment of NDI was represented by using a selective antagonist to block the AVPR2. AVPR2 antagonists such as OPC31260 and tolvaptan caused polyuria and decreased the urine osmolalities of Sprague-Dawley rats to 1000 to 300 mOsM [98,99,100]. In rats, OPC-31260 induced a conspicuous reduction in AQP2 mRNA and in AQP2 protein in the kidney [98,99]. Continuous subcutaneous infusion of tolvaptan using minipumps produced a marked decrease in the expression of AQP2, AQP3, β-subunit of the epithelial sodium channel (ENaC), and γ-ENaC. Animals infused with tolvaptan reduced their total collecting duct water reabsorption by roughly 50%, which was similar to the percent decrease in AQP2 abundance [104].

Overall, the availability of a valid animal model represents a remarkable tool to develop specific and effective pharmacological strategies for the treatment of X-NDI.

## 4. Current Conventional Treatment of Congenital NDI

Current approaches for the treatment of congenital NDI aim to limit the urine output rather than acting at a causative level. The main strategy for handling NDI is to replace the urinary water loss by adequate fluid supply in combination with a low-salt and low-protein diet to minimize the obligatory water excretion. NDI standard therapy includes the use of diuretics and nonsteroidal anti-inflammatory drugs (NSAIDs) and can only partially reduce polyuria. 

Thiazide diuretics effectively reduce urine output when associated with a very low sodium-restricted diet [105]. 

Potassium sparing agents such as amiloride might have an additive effect with thiazide diuretics via a mechanism likely involving the inhibition of potassium loss induced by thiazides [106].

Diuretics in NDI patients reduce the urine output by promoting the reabsorption of sodium and water in the proximal tubule, thus delivering less water to the collecting ducts [39]. NSAIDs such as Ibuprofen and indomethacin improve urinary concentration defects in NDI patients in which urine output can be reduced by 25% to 50% [107,108], and the combination with Hydrochlorothiazide has an additive effect [106,108]. Single-drug therapies show lower efficacy and are therefore not preferred [32,103]. Recently, Dayal et al. investigated the effect of oral administration of indomethacin (0.75 to 1.2 mg/kg/day) three times a day for a mean duration of three years in two children with NDI [109]. Remission occurred in both patients in terms of achieving a normal fluid balance and body growth, and, in one patient, the drug was withdrawn after two years. The treatment was well tolerated, and no side effects were noted. The mean duration of follow-up was 6.5 yrs. However, this observation needs to be tested on a larger number of patients.

In patients who cannot tolerate indomethacin, selective inhibitors of cyclooxygenase-2 (COX2) might be helpful. Caution in using indomethacin and selective COX-2 inhibitors in NDI is required, as their administration can potentially lead to the acute deterioration of renal function in dehydrated patients [39]. The experience with long-term use of cyclooxygenase inhibitors as single agent therapy is limited [103,104,105,106].

Although these therapeutic approaches improve NDI symptoms, the urine concentrating defect is still considerable, representing a serious problem for the patient’s quality of life.

## 5. Possible Therapeutic Strategies to Cure Congenital NDI

### 5.1. Chemical Chaperones

A possible therapeutic approach to rescue the plasma membrane expression of functional misfolded mutant proteins is the use of chemical chaperones to promote escape from the endoplasmic reticulum. Since several AVPR2/AQP2 mutations do not lead to a complete loss of function, different molecules able to re-establish proper protein folding have been analyzed. In contrast, for those AVPR2/AQP2 mutations caused by deletions, insertions, splicing, or rearrangements, it is impossible to consider this therapeutic option. In those cases, gene therapy may eventually become the only possible cure; however, at present, all gene-therapeutic approaches lack safety and efficiency. Another strategy to correct mutant genes is the genome editing of somatic tissue or embryos, but the ethics divide scientists [110]. Therefore, it is difficult to predict when, or even if, these treatments will become a reality.

The AVPR2 rescue has been attempted with limited results using chemical chaperones that, in an unspecific way, aid protein folding such as glycerol and dimethylsulfoxide (DMSO) [52,111].

Chemical chaperones were also tested to correct aberrant folding of AQP2 in autosomal recessive NDI (Figure 3). In CHO and MDCK cells, glycerol, trimethylamine N-oxide (TMAO), and DMSO induced the redistribution of AQP2 mutants from ER to plasma membrane fraction [76]. Another tested molecule is the heat shock protein 90 (Hsp90) inhibitor 17-allylamino-17-demethoxygeldanamycin (17-AAG). Hsp90 is a ‘molecular chaperone’ ER-resident/cytoplasmic protein that aids the proper folding of proteins and promotes the ER-associated degradation pathway (ERAD) of several aberrantly folded proteins [111]. 17-AAG partially corrected NDI in conditional AQP2-T126M knock-in mice, with the partial rescue of defective AQP2-T126M cellular processing [100].

Regarding X-NDI, to overcome the extensive intracellular retention of a functional AVPR2, small cell-permeable molecules able either to rescue AVPR2 expression at the plasma membrane (AVPR2 antagonists) or to activate AVPR2 in the ER (AVPR2 agonists) have been developed.

### 5.2. Nonpeptide AVPR2 Antagonists: Pharmacological Chaperones

Nonpeptide vasopressin receptor antagonists commonly are named vaptans, where vap stands for vasopressin and tan for antagonists [112]. These compounds mimic the structure of AVP and interact with the receptor binding pocket for AVP [113].

Nonpeptide AVPR2 antagonists are lipophilic cell-permeable molecules that can enter the cell to bind class II mutant AVPR2 in the ER and stabilize their conformation.

Once the ER quality control machinery has been bypassed, mutant AVPR2 can exit the ER, achieve mature glycosylation in the Golgi, and localize at the plasma membrane, where high levels of AVP can displace the antagonist and activate the mutant receptor.

Similarly to ER-resident molecular chaperones, these cell-permeable antagonists are called pharmacological chaperones [114]. The AVPR2-selective antagonists SR121463 (satavaptan) [115], VPA985 (lixivaptan) [115], OPC41061 (tolpavtan), OPC31260 (mozavaptan) [111], the AVPR1a antagonist SR49059 (relcovaptan) [57,111,116], and the nonselective AVPR1a/AVPR2 antagonist YM087 (conivaptan) [117] promoted both the sufficient maturation and membrane expression of AVPR2 mutants and their ability to respond to AVP.

The usefulness of a nonpeptide AVPR2 antagonist has been confirmed by a small-scale clinical trial, showing that, in five X-NDI patients with three different AVPR2 mutations, SR49059 had fast-beneficial effects on urine volume and osmolality [117]. Unfortunately, the clinical development of SR49059 has been interrupted during the course of these studies because of a possible interference with the cytochrome P450 metabolic pathway [117]. In a recent study, Smith et al. described an ultra-high-throughput homogeneous cell-based assay with a cAMP detection system to identify pharmacological chaperones of AVPR2 in cells expressing a mutated AVPR2 misfolded protein causing X-NDI [118]. Currently, most pharmacological chaperones possess intrinsic antagonist activity because they were identified using methods initially aimed at discovering such functions. The next step will be to determine whether these compounds act as agonists or as antagonists in cells expressing the wild-type AVPR2 protein. Those that do not have antagonistic characteristics could be interesting compounds for therapeutic intervention [118].

Despite the success of nonpeptide AVPR2 antagonists in restoring receptor plasma membrane expression, there are some limitations regarding their use and development.

First, the effects of the AVPR2 antagonist depend on the type and the location of the AVPR2 mutation [114]. The second problem is their affinity for AVPR2, as the displacement of the antagonist by AVP is inversely correlated with its affinity for the AVPR2.

Therefore, the most efficient functional rescue depends on a balance between the ability of a compound to promote the cell surface expression of the mutant receptor and the possibility that it be displaced by a natural or synthetic agonist for receptor activation [104,112]. Additionally, it has to be considered that, physiologically, the binding of AVP to AVPR2 induces the internalization and rapid degradation of the receptor by the beta-arrestin-MAPK-pathway [119], thus counteracting the effect of any potential rescuing molecule.

### 5.3. Nonpeptide AVPR2 Agonists

Some cell-permeable agonists are able to bind AVPR2 mutants trapped in the endoplasmic reticulum, but, rather than stabilizing their conformation, they directly activate these intracellular AVPR2 mutants by signaling to pre-formed receptor-G protein-adenylate cyclase complexes. The subsequent cAMP production will then activate PKA, leading to AQP2 phosphorylation and plasma membrane expression, thereby attenuating the NDI phenotype [114].

AVPR2 nonpeptide agonists are small molecules, characterized by relatively high hydrophobicity that allows them to pass cell membranes, thereby facilitating an efficient uptake by the gut compared to ‘classical’ peptide agonists such as DDAVP [114]. Agonists OPC51803, VA999088, and VA999089, but not AVP, activated six out of seven AVPR2 mutants within the ER, and induced AQP2 translocation to the apical membrane [120].

Moreover, OPC51803 showed a significant antidiuretic action after single and multiple oral dosing in both AVP-deficient Brattleboro and normal rats [121].

Jean-Alphonse et al. [122] identified nonpeptide AVPR2 agonists that also had positive re-folding effects: MCF14, MCF18, and MCF57. These compounds promoted the maturation and membrane rescue of L44P, A294P, and R337X AVPR2 mutants and restored a functional AVP-dependent cAMP signal. Contrary to pharmacochaperone antagonists, MCFs directly activate a cAMP signal. In addition, unlike AVP, these molecules displayed original functionally selective properties (biased agonism) toward the AVPR2, being unable to trigger receptor internalization through the beta-arrestin-MAPK-pathway [122]. In conclusion, the use of nonpeptide AVPR2 agonists shows different advantages: high selectivity, displacement of the non-peptide compounds by endogenous AVP is not required, and proteasome degradation of the ER-trapped receptors is not increased upon intracellular activation [120].

### 5.4. Bypassing AVPR2 Signaling

In the last few years, several studies highlighted, at least in principle, the possibility to promote urine concentration in NDI patients by circumventing defective AVPR2 signaling and restoring proper AQP2 expression at the apical plasma membrane (Figure 2).

The strategies currently investigated for bypassing the AVPR2 signaling pathway can be divided into two groups:

- Cytosolic cAMP elevation: activation of other G protein-coupled receptors (GPCRs) coupled to Gs/adenylyl cyclase expressed in the collecting duct (CD) principal cells; the inhibition of phosphodiesterases (PDE).
- Activation of cAMP-independent pathways.

#### 5.4.1. Cytosolic cAMP Elevation

Several studies suggest the possibility to restore proper salt and water homeostasis by activating other GPCRs expressed in the same renal cells expressing defective AVPR2. 

*β3-adrenoreceptor*—In a recent work, we demonstrated that β3-adrenoreceptor (β3-AR) is expressed in most of the nephron segments that also express the AVPR2, including the thick ascending limb and the cortical and outer medullary collecting duct [123].

Ex vivo experiments in mouse kidney tubules showed that β3-AR stimulation with the selective agonist BRL37344 increased intracellular cAMP levels and promoted the accumulation of the water channel aquaporin 2 at the apical plasma membrane in the collecting duct and the activation of the Na-K-2Cl symporter in the thick ascending limb. To confirm this observation, we showed that the phenotype of β3-AR-KO mice is characterized by significantly increased urine excretion of water, sodium, potassium, and chloride. 

Moreover, single i.p. injection of BRL37344 in AVPR2-KO mice (X-NDI mice [103]) greatly reduced the diuresis and increases urine osmolality, supporting the notion that, in vivo, β3-AR agonism triggers AVP-independent antidiuresis [123].

*Secretin receptor*—Previous papers have suggested that the secretin receptor (SCTR) is expressed in the kidney medulla [124,125].

More recently, we demonstrated that the SCTR is expressed at the basolateral membrane of AQP2-expressing CD principal cells in mice and humans. Based on this, we analyzed the effect of secretin (SCT) ex vivo on kidney slices and in vivo in AVPR2-KO mice (X-NDI mice [103]) [126]. We provided compelling evidence that SCT induces a dose-dependent rise in intracellular cAMP concentrations in CD tubule suspensions and promotes AQP2 apical expression in freshly isolated kidney slices from control and AVPR2-KO mice. In addition, chronic infusion of SCT increases AQP2 abundance but not its apical expression in AVPR2-KO mice. It is of note that, in SCT-infused X-NDI mice, a single injection of fluvastatin (see below), a drug that induces AQP2 membrane accumulation in wt C57BL/6 mice [127], promotes AQP2 membrane expression and greatly improves the urine concentration ability, reducing urine output by nearly 90% and doubling urine osmolality [126].

*Calcitonin receptor*—Previous works with calcitonin (CT) showed a possible AVP-like effect on electrolyte and renal water reabsorption [128,129]. The renal distribution of CT receptors differs among species [130,131]. In humans, CT stimulates adenylyl cyclase activity in thick ascending limbs and in cortical and medullary collecting ducts [132], while, in rats, it acts in the cortical collecting ducts, distal convoluted tubules, and thick ascending limbs [131,132,133,134]. Overall, the similar localization of both CT and AVPR2 receptors in the same nephron segments is consistent with the notion that CT might exert an AVP-like effect on water reabsorption [129]. Bouley et al. investigated the effect of CT on AQP2 trafficking in vitro, ex vivo, and in vivo using LLC-PK1 kidney epithelial cells, kidney slices, and CT-infused AVP-deficient Brattleboro rats, respectively [135]. CT increased intracellular cAMP in AQP2-expressing LLC-PK1 cells expressing endogenous CT receptors, promoting AQP2 membrane accumulation. In addition, immunocytochemistry on Brattleboro rat kidneys showed a CT-induced increase of AQP2 membrane accumulation in cells from cortical collecting ducts, in parallel with a significant but transient reduction in urine volume and a two fold increase in urine osmolality, when compared with control rats [135]. As concluded by the same authors, additional studies are required to determine whether CT might also be beneficial to patients with X-NDI.

*Prostaglandin receptors*—The prostaglandin receptor subtype 4 (EP4 PGE2)is a Gs-coupled receptor expressed in mouse and rat inner medullary collecting duct (IMCD) cells [103,136]. Using the first viable mouse model of X-NDI, Li and colleagues demonstrated that ONOAE1-329, a compound that selectively activates the EP4 PGE2 receptor subtype, was highly effective in ameliorating all major manifestations of X-NDI, including polyuria and dilatation of the renal pelvis. In addition, prolonged treatment of X-NDI mice with ONOAE1-329 significantly increased renal AQP2 levels, probably due to EP4 receptor-mediated elevations of cAMP levels in kidney collecting duct cells [103]. Other agonists, specific for EP2 (butaprost) and EP4 (CAY10580) receptors, were shown to increase AQP2 trafficking [137], although the two mechanisms of action are likely to be different, since only EP2 stimulation increased cAMP in MDCK cells [137]. In the same study, butaprost was able to reduce urine output and increase urine osmolality by up to 65% in a rat model of X-NDI. 

However, it is important to note that intrarenal PGE2 infusion promotes diuresis [138], probably via the activation of other PGE2 receptor subtypes mediating the inhibition of salt and water absorption along the nephron [139]. The inhibition of these latter effects of PGE2 by indomethacin, which reduces tissue PGE2 levels via the nonselective inhibition of cyclooxygenase 1 and 2, is thought to contribute to the ability of this drug to reduce urine output in X-NDI patients [140]. Therefore, selective PGE receptor antagonism may represent an efficient mean of controlling water excretion.

The binding of PGE2 to EP3 expressed in the kidney [141] counteracts the AVP-induced increase of osmotic water permeability in the renal CD by inactivating the adenylate cyclase, thus resulting in an increase of actin polymerization, via Rho activation, that prevents AQP2 trafficking to the apical membrane [140].

Every effort should be made to develop other PGE receptor inhibitors targeting other PGE receptor isoforms. 

Another potential strategy to increase cytosolic cAMP levels is the inhibition of phosphodiesterases (PDE). A study showed that Rolipram, a selective PDE4 inhibitor, increases urine osmolality in a hypercalcemia-induced NDI mice model [81]. Rolipram increases cAMP content in the papillae, along with the phosphorylation and translocation of AQP2. Although PDE3 and PDE5 are also thought to be expressed in the collecting ducts [142], inhibitors of these PDEs were ineffective. In a clinical study, however, Rolipram treatment of two male patients with NDI due to AVPR2 mutations did not induce any relief of symptoms [143].

#### 5.4.2. Activation of cAMP-Independent Signaling Cascades

Different strategies have been described in the literature to bypass the AVPR2-elicited pathway in a cAMP-independent way: activation of the cyclic guanosine monophosphate (cGMP) pathway (NO, Sidenafil), epidermal growth factor receptor (EGFR) inhibition (Erlotinib), AMP-activated protein kinase (AMPK) activation (Metformin), Statins, and peroxisomal proliferetor-activated receptor subtype γ (PPAR-γ) agonists (Rosiglitazone). 

*cGMP signaling*—In addition to the canonical cAMP-induced pathway, the activation of the cGMP path way can modulate AQP2 trafficking [144,145,146].

Nitric oxide (NO) donors such as sodium nitroprusside (SNP) and NONOate, as well as the nitric oxide synthase (NOS) substrate l-arginine, induced AQP2 cell-surface localization by a cAMP-independent and cGMP-dependent pathway in rat kidney slices and LLC-PK1 cells expressing AQP2 [144]. In addition, atrial natriuretic peptide (ANP), infused in rats, stimulated AQP2 membrane insertion [146]. The mechanism by which cGMP induces AQP2 trafficking is still unclear. Bouley et al. demonstrated that purified AQP2 COOH tail can be phosphorylated by PKG [144]. However, neither the possibility that PKG phosphorylates PKA nor that cGMP directly activates PKA can be reasonably ruled out. In the absence of functional AVPR2, however, AQP2 expression levels are also downregulated because cAMP stimulates AQP2 transcription through a cAMP-responsive element in its promoter [147,148,149,150]. Boone and colleagues used a mouse cortical collecting duct cell line (mpkCCD) to determine whether the activation of the cGMP-signaling pathway might, not only induce AQP2 translocation, but also increase AQP2 expression. The authors found that ANP, l-arginine, and 8-Br-cGMP induced the translocation of AQP2 but did not significantly increase AQP2 expression in mpkCCD cells [151]. Therefore, cGMP activators should be combined with other agents that stimulate AQP2 transcription to improve their beneficial effect in NDI treatment.

The elevation of intracellular cGMP could also be achieved by cGMP PDE inhibitors. Several PDE isoforms are expressed along the nephron such as the cGMP-sensitive PDE5 and the cAMP/cGMP sensitive PDE1 [142]. The acute exposure of both LLC-PK1 cells expressing AQP2 and rat kidney slices to PDE5 inhibitors sildenafil citrate (Viagra) and 4-{[3′,4′-methylene-dioxybenzyl]amino}-6 methoxyquinazoline (MBMQ) increased cGMP and resulted in AQP2 accumulation at the plasma membrane. Importantly, AQP2 apical expression was observed also in kidneys from Brattleboro rats injected with sildenafil [145]. Sildenafil was also shown to reduce polyuria and increase urine osmolality in rats with lithium-induced NDI [152]. Recently, Assadi and Sharbaf described a case of a young male patient with X-NDI treated with sildenafil and showing a substantial decrease in urine output and increased urine osmolality compared with conventional treatment [153]. Strikingly, they also detected an increase of urinary excretion of AQP2, suggesting that cGMP can also increase AQP2 expression. This finding is inconsistent with those reported by Boone et al. in mice [151]. The minimal activity of the AVPR2 mutation in this patient may also play a role in the beneficial effect of sildenafil [153].

*EGFR inhibitor*—A large amount of evidence suggests that EGF tonically inhibits the effect of AVP [154] by decreasing AQP2 phosphorylation at Ser-256 [155]. Recently Nomura et al. found, by a high-throughput chemical screening assay, that AG-490, an inhibitor of both EGF receptor (EGFR) and JAK-2 kinase, increased AQP2 membrane expression and water reabsorption in rats [156]. Cheung and colleagues demonstrated that the Food and Drug Administration approved EGFR inhibitor, Erlotinib, increased AQP2 membrane accumulation in the collecting duct principal cells, greatly reduced urine output, and increased urine osmolality in mice with lithium-induced NDI [155]. Although Erlotinib mimicked AVP-mediated AQP2 phosphorylation, they found that this was independent in the cAMP, cGMP, and PKA activation pathways and did not result in the phosphorylation of the PKA substrate cAMP response element binding protein (CREB) [157], which is pivotal in the regulation of AQP2 transcription. The authors hypothesized that an unidentified kinase, responsible for AQP2 phosphorylation, is activated upon EGFR inhibition [155]. Considering that X-NDI is characterized by both the lack of AQP2 membrane expression and AQP2 severe downregulation, it is likely that a combination of therapies will be necessary, along with EGFR inhibitors, to hypothesize the introduction of these molecules in the therapy of NDI.

*Metformin*—Metformin is an oral antidiabetic drug that stimulates both AMPK catalytic subunits [158]. AMPK is a serine/threonine kinase stimulated by osmotic stress and hypoxia, and recently it has been shown to be expressed in the kidney medulla, where it can phosphorylate both AQP2 and the urea transport UT-A1, thus promoting water and urea reabsorption in rat inner medullary collecting duct cells [159]. Efe et al. demonstrated that Metformin improved urine concentrating ability in rats treated with the AVPR2 blocker tolvaptan, as well as in AVPR2-KO mice [160]. To explain this effect, the author suggested that, under physiological conditions, the vasopressin-dependent activation of PKA suppresses AMPK activity, so any phosphorylation/activation of AQP2 or UT-A1 by AMPK is minimal. The authors speculated that, in X-NDI, this tonic suppression is absent and that AMPK might be responsible for a novel compensatory stimulatory pathway. Furthermore, in the absence of AVPR2, AMPK upregulated AQP2 and UT-A1 at a protein level. Collectively, these data suggest that Metformin could represent a novel approach for treating X-NDI.

*Statins*—Statins competitively inhibit hepatic 3-hydroxy-3-methylglutaryl-coenzyme A reductase [161], reduce plasma total and low-density lipoprotein cholesterol levels, and have been approved for the treatment of hypercholesterolemia in humans. Many recent papers have highlighted a new pleiotropic effect of statins in promoting AVP-independent apical localization of AQP2 in renal cells in vitro and in vivo [127,162,163,164,165]. This additional effect is independent of cholesterol homoeostasis and depends on the depletion of mevalonate-derived isoprenoid intermediates of sterol synthetic pathways, i.e., farnesylpyrophosphate and geranylgeranylpyrophosphate [127,166]. 

We previously reported that lovastatin can promote the accumulation of AQP2 at the apical membrane of renal cells in vitro by inhibiting AQP2 constitutive endocytosis [162]. Later, we demonstrated that fluvastatin increased water reabsorption in an AVP-independent manner in wild-type mice [127]. Fluvastatin caused changes in the prenylation status of key proteins regulating AQP2 trafficking in collecting duct cells. We identified RhoA, involved in the actin cytoskeleton network, and Rab5, implicated in clathrin-coated vesicles (CCV)-mediated endocytosis, as possible candidates, the reduced prenylation of which might result in the accumulation of AQP2 at the plasma membrane [127]. Interestingly, we reported that the combination of fluvastatin and secretin reduced urine output by nearly 90% and doubled urine osmolality in AVPR2-KO mice [126]. 

In accordance with these data, simvastatin was reported to decrease AQP2 endocytosis and increase urine-concentrating ability in Brattleboro rats [164]. 

We also studied the effect of statins on AQP2 trafficking in an adult male NDI patient carrying an inactivating mutation of the AVPR2 [165]. AQP2 membrane expression in the kidney was monitored by measuring the urinary excretion of AQP2 (u-AQP2) after treatment with 40 and 80 mg/day fluvastatin for 90 days. We found that u-AQP2 increased in a time- and dose-dependent manner. However, at this drug dosage, increased uAQP2 was not accompanied by a reduction of diuresis and an increase of urine osmolality [165]. In another study, we found that Simvastatin treatment induced a rapid and significant increase of uAQP2, reduced the 24-h diuresis, and increased urine osmolality in a cohort of hypercholesterolem subjects with preserved renal function. These effects were also maintained in patients chronically treated with statins for at least one year [167]. The preliminary findings showed that statins treatment (atorvastatin and rosuvastatin) was also beneficial in preventing lithium-induced NDI [168]. A randomized Phase 2 clinical study is ongoing to confirm the results (https://clinicaltrials.gov/ct2/show/NCT02967653). All data strongly suggest that statins may enhance the efficacy of the current pharmacological treatment of patients with X-NDI.

*Rosiglitazone*—Thiazolidinediones are peroxisomal proliferator-activated receptor sub-type γ (PPAR-γ) agonists used as oral antidiabetic drugs. Unfortunately, one side effect of one of these drugs, rosiglitazone (RGZ), is the tendency to lead to fluid retention [169], causing edema and congestive heart failure [170] by a not fully understood mechanism likely involving increased salt and water reabsorption in the kidney. Many works tried to demonstrate the involvement of RGZ in modulating the expression of the main sodium transporters and water channels in the kidney [171,172,173]. Recently, we investigated in vitro whether RGZ may activate a transduction pathway that facilitates AQP2 membrane accumulation in renal cells. We found that RGZ induces a dose-dependent translocation of AQP2 exocytic vesicles to the apical plasma membrane. This event is independent on intracellular cAMP elevation, PKA activation, and AQP2 phosphorylation but is concomitant with Ca^2+^ influx, which is likely mediated by RGZ-induced TRPV6 channel activation. Importantly, this effect suggests an unexplored application of RGZ in the treatment of pathological states characterized by impaired AQP2 trafficking at the plasma membrane as X-NDI [174].

## Figures and Tables

**Table 1:** Overview and classification of mutations causing nephrogenic diabetes insipidus (NDI) as reported by HGMD^®^ Professional 2017.3 as of September 2017.

Gene/Mutation Type	Location	Disease	Number of Mutations
**AQP2**			
Missense/nonsense	12q12–q13	Autosomal recessive NDI	46
	12q12–q13	Autosomal dominant NDI	4
Splicing	12q12–q13	Autosomal recessive NDI	4
Small deletions	12q12–q13	Autosomal recessive NDI	3
	12q12–q13	Autosomal dominant NDI	6
Small insertions	12q12–q13	Autosomal dominant NDI	1
	12q12–q13	Autosomal recessive NDI	1
**TOTAL**			65
**AVPR2**			
Missense/nonsense	Xq28	X-linked NDI	166 (1 partial)
Splicing	Xq28	X-linked NDI	4
Small deletions	Xq28	X-linked NDI	52
Small insertions	Xq28	X-linked NDI	19
Small indels	Xq28	X-linked NDI	5
Gross deletions	Xq28	X-linked NDI	23
Gross insertions	Xq28	X-linked NDI	1
Complex rearrangements	Xq28	X-linked NDI	4
**TOTAL**			274
